# Department of Surgery

**Published:** 1901-07

**Authors:** W. E. A. Wyman, J. A. Sloan, Robert Jay

**Affiliations:** Milwaukee, Wis.; San Francisco, Cal.; West Liberty, Ia.


### DEPARTMENT OF SURGERY.

By W. E. A. Wyman, M.D.V., V.S.,
MILWAUKEE, WIS.

J. A. Sloan, B.Sc., M.D.V.,
SAN FRANCISCO, CAL.

Robert Jay, M.D.V.,
WEST LIBERTY, IA.

### Hoof Diseases.

(Continued from page 377.)

### Hemorrhagic Pododermatitis.

In those animals predisposed to pododermatitis hemorrhagica the prognosis is, as a rule, unfavorable, while the result of mechanical insults are more favorable ; at the same time the extent, the seat, the duration, and the intensity must first be considered before a final favorable verdict is issued. Remembering the fact that in this disease more or less changes have taken place in the walls of the bloodvessels, a considerable length of time is essential for a cure. The least time, according to the writer's experience, required for the cure of hemorrhagic pododermatitis is four weeks. In chronic cases the prognosis is invariably unfavorable for obvious reasons.

In the treatment of hemorrhagic pododermatitis the general rules laid down for serous pododermatitis hold good. Thus rest is the *conditio sine qua non.* Of course, this will, or, rather, must be granted, as the animal is too lame to work ; but for a successful termination of the treatment this rest must be extended over the whole period, as the lameness would certainly reappear if the animal is put to work too soon. In the earliest stages, in violent cases only, jugular phlebotomy is indicated. As regards scarification of the coronary region and bleeding from the toe, the same opinion is held by the writer as expressed under serous pododermatitis. Therefore, in the earliest stages, those which the veterinarian usually is not called upon to treat, the application of cold in some form or other, preferably continuous irrigation, is indicated. (See Serous Pododermatitis.) But after the state has existed for several days heat and moisture only are of use ; in the shape of aseptic cataplasms here, also, for reasons previously given, the heels, coronary region, and hollow of the heels must be dressed with some lardaceous agent to prevent excessive maceration of the parts and possibly coronary contractions. (See Serous Pododermatitis.) In order to hasten subsequent resorption a light cantharides blister to the coronary region is of value. As regards the removal of the shoe, the same holds true as stated under Serous Pododermatitis ; in other words, it is indicated in some cases and positively wrong in others, as, for instance, in hoofs with a flat sole or in the weak-heeled, acute-angled, or long-toed hoof. A bar shoe with a leather pad or a Climax rubber pad are of use as soon as the animal is able to go to work.

### Suppurative Pododermatitis.

An acute or chronic, superficial or deep, circumscribed or diffused septic inflammation of the pododerm, of variable intensity, preferably affecting the posterior section of the hoof.

The direct cause of suppurative pododermatitis is pus-producing bacteria, and of these the staphylococcus pyogenes aureus and streptococcus pyogenes are apparently the most frequent ones, while the staphylococcus pyogenes albus and citreus also are of interest. In order to do this important subject justice superficial suppurative pododermatitis will be discussed individually.

*Superficial Suppurative Pododermatitis.* In this form of pododermatitis the phylloid stratum, viz., the papillary layer and stratum mucosum, are infected with pus-producing bacteria, and exhibit a purulent exudate.

Any injury to the pododerm or parts immediately surrounding it may lead to an infection.

(To be continued.)

---

The Pennsylvania Legislature, without one dissenting voice in the Senate or House, granted $10,000 to the State Live-stock Sanitary Board for laboratory work during the next two years. This sum is in excess of the grant two years ago.

Dr. J. M. Gibb, government inspector of stock at Jamaica, states that they have a marvellously healthy climate, but that they suffer more or less from some serious disorders among the cattle. Tick fever, diphtheritic stomatitis among calves, red water, principally among imported cows and bulls. In sheep swamp fever is the principal trouble, due to the ravages of fluke and other worms. Among the porcine tribe swine-cholera, while among horses adenitis, suppurative dermatitis, ulcerative dermatitis, divide his attention.

